# Immuno-haematologic and virologic responses and predictors of virologic failure in HIV-1 infected adults on first-line antiretroviral therapy in Cameroon

**DOI:** 10.1186/2049-9957-3-5

**Published:** 2014-01-30

**Authors:** Henry D Meriki, Kukwah A Tufon, Mbunkah H Afegenwi, Bernard A Nyindem, Pascal N Atanga, Damian N Anong, Fidelis Cho-Ngwa, Theresa Nkuo-Akenji

**Affiliations:** 1 Department of Microbiology and Parasitology, University of Buea, P.O. Box 63, Buea, Cameroon; 2 BioCollections Worldwide Inc. Regional Office, Buea, Cameroon; 3 Regional Delegation of Public Health, Southwest Region, Cameroon; 4 Faculty of Science Clinical Diagnostic Laboratory, University of Buea, Buea, Cameroon

**Keywords:** Immuno-haematologic, Predictors, Virologic failure, Antiretroviral therapy

## Abstract

**Background:**

Contemporary data on the immunologic, haematologic and virologic responses and predictors of virologic failure after initiation of free antiretroviral treatment in Cameroon are needed to evaluate the current treatment-monitoring algorithm and to complement efforts to scale-up and improve on the management of HIV infections.

**Methods:**

This was a cross-sectional study conducted between October 2010 and June 2012. A total of 951 participants aged 18–74 years were recruited from selected approved HIV treatment centres of the Northwest and Southwest regions. This comprised 247 males and 704 females. Demographic, self-reported risk behaviours and socioeconomic data were obtained using a structured questionnaire. Full blood and CD4 + T-cell counts were done using standard automated techniques. Determination of viral load (VL) was done using Abbott Real*Time* HIV-1 *m*2000™ system. Data was analysed using SPSS version 17. The statistical significance level was P < 0.05.

**Results:**

The median duration of antiretroviral therapy (ART) was 24 months. The population mean CD4 + T-cell count was 255.3 cells/μL [95% CI, 236.8 – 273.9]. Overall, 45.9%, 43.8% and 10.2% of the participants had CD4 + T-cell counts of < 200 cells/μL, 200–499 cells/μL and > 500 cells/μL respectively. Anaemia was present in 26.2% of the participants with 62.3%, 25.7% and 12% described as mild, moderate and severe anaemia respectively. Virologic failure occurred in 23.2% of the participants with 12.3% having VL > 10,000 RNA copies/mL. Meanwhile 76.8% of patients attained adequate viral suppression with 40.8% having undetectable viral load. The age group 18–29 years (p = 0.024), co-infection with tuberculosis (p = 0.014), anaemia (p = 0.028) and distance from the treatment centre (p = 0.011) independently predicted virologic failure.

**Conclusion:**

The majority of the participants achieved adequate viral suppression after ≥ 6 months of ART. Despite these favourable immuno-haematologic and virologic outcomes, the National AIDS Control Program should step-up efforts to improve on antiretroviral drug distribution, as well as proper assessment and management of anaemia, foster early diagnosis and treatment of tuberculosis and enhance treatment adherence counselling especially in younger patients.

## Multilingual abstracts

Please see Additional file 1 for translations of the abstract into the six official working languages of the United Nations.

## Background

The introduction of free antiretroviral therapy (ART) has substantially improved on the health status of HIV infected patients. Although high levels of adherence have been reported in small-scale HIV programs in Sub-Saharan Africa [1], more challenges arise as these programs scale-up particularly in countries with a growing burden of HIV and tuberculosis (TB), and limited healthcare management facilities [2]. Rapid scale-up of antiretroviral therapy is accompanied by an increasing risk of ART failure resulting from HIV drug resistance and this is a major obstacle to successful ART in HIV-infected patients [3]. ART failure may result in progression to AIDS characterized by immunological and haematological complications and opportunistic infections [4] with increased risk of morbidity and mortality.

Generally, clinical response to ART in resource-limited settings is monitored with CD4 + T cell counts and some hematologic indices [2,5]. For successful outcomes and the amelioration of ARV treatment, the challenge is the effective delivery of ART with the aim of attaining high treatment success rates [5]. However, the assessment of viraemia, which gives a glaring picture of disease progression as well as response to ART [6], is not part of this clinical monitoring in our setting due to its unavailability and cost [2]. With appropriate adherence to ART, it is expected that viral load drops to undetectable levels after ≥ 6 months of ART [7], which greatly reduces the likelihood of sexual transmission [8], morbidity and mortality among HIV infected patients. Therefore, identifying risk factors predicting treatment outcomes would be a reliable alternative for achieving a high treatment success rate. These identified risk factors could be useful for rendering effective support and services to patients at risk of treatment failure before or during treatment.

HIV prevalence among adults between the ages of 15 and 49 years in Cameroon is 4.3% [9]. The Northwest (NW) and the Southwest (SW) regions with over 1.8 million and 1.3 million inhabitants respectively [10] shoulder the highest burden of HIV in the country with prevalence of 6.3% and 5.7% respectively [9]. The prevalence of HIV in the NW region has been steadily high, even though it declined from 8.5% recorded in 2010 [11]. The NW and SW regions have 16 and 15 approved treatment centres respectively that take care of over 16,000 and 12,000 patients on ART respectively [11]. Therefore, contemporary data on immunologic, haematologic and virologic responses after the initiation of free antiretroviral therapy in Cameroon are needed to inform and complement efforts to scale-up and improve on the management of HIV infection. In this study, we assess the immuno-haematologic and virologic status as well as factors associated with virologic failure of HIV-1 infected patients on ART in NW and SW regions of Cameroon.

## Methods

This was a cross-sectional, hospital-based study involving HIV-infected patients enrolled on high active antiretroviral therapy (HAART), treated between October 2010 and June 2012. Participants were consecutively recruited from the approved HIV treatment Centres of Buea and Limbe Regional hospitals, the management units of the Tiko Central Clinic and Kumba District hospital of the Southwest region; the HIV treatment centre of the Bamenda Regional hospital and the management unit of St. Theresa Catholic Medical Centre of the Northwest region. A nurse assigned to the study obtained demographic and self-reported risk behaviour and socioeconomic data with a standardized questionnaire [12] after obtaining participant’s consent.

Two blood samples (2 × 5 mL) were collected in ethylene diamine tetra-acetate vaccutainer tubes by a technician from each participant. One tube was centrifuged (at 1100 *g* for 20 minutes) and plasma aliquoted and stored at −20°C until needed. The second sample was used for full blood count analysis using the auto haematology analyser (BC-2800, Mindray Bio-Medical Electronics, Shenzhen, China) and CD4 + T-cell count (BD Biosciences FACSCount™, New Jersey, USA) following manufacturers’ instructions. Anaemia was defined as haemoglobin concentration < 11 g/dL [13]. Anaemia was further categorised as mild (9.6 – 10.9 g/dL), moderate (8 – 9.5 g/dL) and severe (< 8 g/dL). Plasma viral load (VL) was determined using the Abbott RealTime HIV-1 m2000™ System (Abbott Molecular Inc. Des Plaines, Illinois, USA) according to the manufacturer’s instructions. Virologic failure was defined as VL > 400 RNA copies/mL after ≥ 6 months of ART while adequate suppression was VL ≤ 400 RNA copies/mL after ≥ 6 months of ART [14].

The data was analysed with SPSS 17.0 (Statistical Package for the Social Sciences, Chicago, Illinois). Continuous variables were compared using the *t*-test. Univariate analysis was performed with Chi-square and only significant associated risk factors were included in a logistic regression model. Odds ratios (ORs) and nominal 95% confidence intervals (CIs) were presented. A two-sided p-value < 0.05 was considered significant for all analyses. Ethical clearance for the study was obtained from Cameroon National Ethics Committee.

## Results

### Description of the study population

Of the 951 participants recruited for the study, 247 were males [mean age: 42.9 years, 95% CI 41.6 – 44.1] and 704 were females [mean age: 38.1 years, 95% CI, 37.4 – 38.9]. Of 951 these participants, 163 were from the NW and 788 from the SW regions. Over 50% of the study participants were between the ages of 30 and 45 years old. The majority (83.6%) of the study participants had a monthly income of ≤ 50,000 XAF (~ ≤ 100 US dollars), 48.9% had attained more than 7 years of education, while 41.7% reported to be currently married.

Most of the participants (88.3%) were receiving treatment at approved treatment centres while 11.7% were treated at the approved management units. The median duration of ART was 24 months [Range: 1–156 months]. Most of the participants (72.9%) were on nevirapine (NVP)-based regimen (17.5% on AZT/3TC/NVP and 55.4% on d4T/3TC/NVP) while 27.1% were on efavirenz (EFV)-based regimen (12.6% on AZT/3TC/EFV, and 14.5% on d4T/3TC/EFV). The prevalence of smoking and alcohol consumption in the study population was 9.1% and 54.3% respectively. Of the alcohol consumers, 48.2% were hazardous drinkers (male > 3 bottles of beer/occasion and females > 2 bottles of beer/occasion), while 16% of the participants both smoked and drank alcohol. Smoking (p < 0.001) and alcohol consumption (p = 0.003) were significantly higher among males than female participants. A total 10.8% of the study participants were co-infected with tuberculosis (Table 1).

**Table 1:** Baseline characteristics of study participants categorized by gender

**Variables Category**	**Overall**	**Male**	**Female**	**P-value**
	**n (%)**	**n (%)**	**n (%)**	
**Socio-demographic**				
**Age group (year):**				
18 – 29	153 (16.1)	19 (12.4)	134 (87.6)	< 0.001
30 – 45	561 (59.0)	144 (25.7)	417 (74.3)	
> 45	237 (24.9)	84 (35.4)	153 (64.6)	
**Marital status:**				
Currently	303 (41.7)	133 (43.9)	170 (56.1)	< 0.001
Never	231 (31.8)	37 (16.0)	194 (84.0)	
Previously	193 (26.5)	18 (9.3)	175 (90.7)	
**Income level (XAF):**				
≤ 50,000*	628 (83.6)	143 (22.8)	485 (77.2)	< 0.001
> 50,000	123 (16.4)	50 (40.7)	73 (59.3)	
**Level of education:**				
≤ 7years of school (low)	368 (48.9)	93 (25.3)	275 (74.7)	0.747
> 7 years of school (high)	384 (51.1)	101 (26.3)	283 (73.7)	
**Region of residence:**				
South West	788 (82.9)	198 (25.1)	590 (74.9)	0.191
North West	163 (17.1)	49 (30.1)	144 (69.9)	
**Management Facility:**				
AMU	111 (11.7)	33 (29.7)	78 (70.3)	0.337
ATC	840 (88.3)	214 (25.5)	626 (74.5)	
**Behavioural**				
**Smoking:**				
Yes	71 (9.1)	48 (67.6)	23 (32.4)	< 0.001
No	708 (90.9)	151 (21.3)	557 (78.7)	
**Alcohol use:**				
Yes	423 (54.3)	126 (29.8)	297 (70.2)	0.003
No	356 (45.7)	73 (20.5)	283 (79.5)	
Hazardous alcohol use^ф^	204 (48.2)	61 (29.9)	143 (70.1)	0.960
Standard alcohol use	219 (51.8)	65 (29.7)	154 (70.3)	
**Alcoholic-smoker:**				
Both	68 (16.0)	46 (67.6)	22 (32.4)	< 0.001
Drinks or smokes	358 (84.0)	82 (22.9)	276 (77.1)	
**Clinical history**				
**TB co-infection:**				
Yes	90 (10.8)	27 (30.0)	63 (70.0)	0.354
No	746 (89.2)	190 (25.5)	556 (74.5)	
**Regimen base:**				
NVP	614 (72.4)	145 (23.6)	469 (76.4)	0.044
EFV	234 (27.6)	71 (30.3)	163 (69.7)	
**Duration of ART:**				
< 12 months	216 (25.2)	57 (26.4)	159 (73.6)	0.888
12 – 36 months	390 (45.6)	103 (26.4)	287 (73.6)	
> 36 months	250 (29.2)	62 (24.8)	288 (75.2)	

SD-standard deviation, XAF- Central African CFA franc, *50,000 XAF = ~ 100 US dollars, AMU-approved management unit, ATC-Approved treatment centre, ^ф^ Hazardous alcohol use (Female < 3 and male < 4 bottles of beer/occasion), EFV-Efavirenz, NVP-Nevirapine.

### Immunologic responses

**Figure 1:**
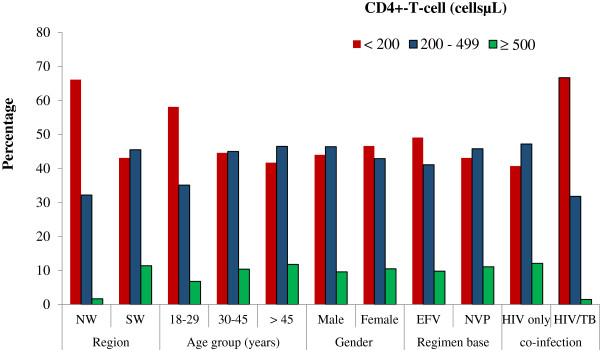
Prevalence of immunologic categories among study participants.

CD4 + T-cell count measurements were performed in 479 of the 951 participants. The population mean CD4-T-cell count was 255.3 cells/μL [95% CI, 236.8 – 273.9]. Mean CD4-T-cell count was significantly higher (p < 0.001) in participants from the SW than NW regions and in the age group > 45 years when compared with those younger (p = 0.018) (Table 2). This was also true for HIV mono-infected (p < 0.001) when compared with HIV/TB co-infected patients. However, there was no significant difference (p = 0.171) in the mean CD4 + T-cell count in patients who were treated with Efavirenz-based and Nevirapine-based regimens. Similarly, CD4 + T-cell count was not significantly different (p = 0.397) between males and females (Table 3). Overall, 45.9%, 43.8% and 10.2% of the participants had CD4 + T-cell counts of < 200 cells/μL, 200–499 cells/μL and > 500 cells/μL respectively. CD4 + T-cell counts of < 200 cells/μL were significantly higher in HIV/TB co-infected cases (p < 0.001) and in patients from the NW region (p = 0.002) (Figure 1).

**Table 2:** Mean CD4 + Tcell count, viral load and haematologic parameters of study participants, grouped by region, age and gender

		**CD4+ T-cell count**		**Log**_**10 **_**viral load**		**Mean haematologic parameters ± SD**				
**Variables Category**		**N**	**Mean ± SD [cells/μL]**	**N**	**Mean ± SD [copies/mL]**	**N**	**Hgb [g/dL]**	**RBC [x10**^**6**^**/mm**^**3**^**]**	**WBC [x10**^**3**^**/mm**^**3**^**]**	**Platelet [x10**^**5**^**/mm**^**3**^**]**
**Region:**										
Northwest		59	174.9 ± 120.1	75	3.32 ± 1.38	99	11.69 ± 2.21	3.42 ± 0.61	4.24 ± 1.61	2.25 ± 1.05
Southwest		420	266.7 ± 213.8	273	3.13 ± 1.31	538	12.07 ± 2.00	3.69 ± 0.62	4.41 ± 1.63	2.36 ± 0.77
**p-value**			**0.001**		**0.275**		**0.094**	**< 0.001**	**0.342**	**0.199**
**Age groups (years)**										
18-29		74	200.1 ± 154.8	75	3.35 ± 1.33^♠^	87	11.42 ± 1.69^†^	3.62 ± 0.53	3.99 ± 1.31^‡^	2.40 ± 0.88
30-45		278	256.8 ± 198.7^ᶲ^	195	3.23 ± 1.34^♠^	371	12.09 ± 2.14	3.69 ± 0.66	4.44 ± 1.79	2.35 ± 0.78
> 45		127	285.8 ± 242.3^ᶲ^	78	2.84 ± 1.24	179	12.15 ± 1.94	3.58 ± 0.60	4.45 ± 1.36	2.32 ± 0.86
**p-value**			**0.018**		**0.033**		**0.012**	**0.124**	**0.049**	**0.732**
**Gender**										
Male		125	241.9 ± 177.9	98	3.13 ± 1.37	158	13.24 ± 2.16	3.93 ± 0.72	4.39 ± 1.55	2.20 ± 0.76
Female		354	260.1 ± 215.9	250	3.19 ± 1.31	479	11.61 ± 1.83	3.56 ± 0.57	4.39 ± 1.65	2.39 ± 0.83
**p-value**			**0.397**		**0.724**		**< 0.001**	**< 0.001**	**0.996**	**0.010**

^♠^ Higher than 30–45 and > 45 years [p < 0.025], ^ᶲ^ higher than 18–29 years [p < 0.037], ^§^ higher than < 12 months [p = 0.03], ^*^ higher than < 12 months and 12–36 months [p < 0.001], ^†^lower than 30–45 years and > 45 years [p < 0.005], ^‡^ lower than 30–45 and > 45 years [p < 0.03].

**Table 3:** Mean CD4 + Tcell count, viral load and haematologic parameters of study participants grouped by ART duration, regimen type and co-infection status

		**CD4+ T-cell count**		**Log**_**10 **_**viral load**		**Mean haematologic parameters ± SD**				
**Variables Category**		**N**	**Mean ± SD [cell/μL]**	**N**	**Mean ± SD [copies/mL]**	**N**	**Hgb [g/dL]**	**RBC [x10**^**6**^**/mm**^**3**^**]**	**WBC [x10**^**3**^**/mm**^**3**^**]**	**Platelet [x10**^**5**^**/mm**^**3**^**]**
**ART duration (Months)**										
< 12		106	168.9 ± 143.8	118	3.60 ± 1.27	135	11.54 ± 2.09^ℓ^	3.58 ± 0.61	4.10 ± 1.49	2.50 ± 0.95
12 – 36		255	266.3 ± 214.1^§^	153	3.07 ± 1.38	294	11.97 ± 1.90^₸^	3.63 ± 0.59	4.33 ± 1.61	2.29 ± 0.77
> 36		130	318.7 ± 220.5^*^	60	2.93 ± 1.05	192	12.56 ± 2.06	3.78 ± 0.66^∞^	4.69 ± 1.75^δ^	2.36 ± 0.77
**p-value**			**< 0.001**		**0.001**		**< 0.001**	**0.008**	**0.004**	**0.054**
**Regimen type**										
EFV-based		163	241.0 ± 186.4	84	3.20 ± 1.37	179	11.91 ± 1.96	3.59 ± 0.59	4.28 ± 1.65	2.48 ± 0.97
NVP-based		297	268.9 ± 219.8	243	3.26 ± 1.30	436	12.12 ± 2.06	3.69 ± 0.63	4.44 ± 1.65	2.31 ± 0.75
**p-value**			**0.171**		**0.683**		**0.241**	**0.087**	**0.287**	**0.024**
**Co-Infection status**										
HIV only		398	274.3 ± 216.6	255	3.18 ± 1.35	574	12.14 ± 2.02	3.71 ± 0.62	4.44 ± 1.67	2.36 ± 0.76
HIV/TB		66	171.1 ± 112.1	42	3.50 ± 1.34	63	10.89 ± 1.86	3.09 ± 0.42	3.89 ± 1.06	2.20 ± 1.22
**p-value**			**< 0.001**		**0.166**		**< 0.001**	**< 0.001**	**0.011**	**0.154**

EFV-Efavirenz, NVP-Nevirapine, ^§^ higher than < 12 months [p = 0.03], ^*^ higher than < 12 months and 12–36 months [p < 0.001], ^ℓ^ lower than 12–36 and > 36 months [p < 0.02], ^⍑^ lower than > 36 months [p = 0.02], ^∞^ higher than < 12 months [p = 0.012], ^δ^ higher 12 months [p = 0.004].

### Haematologic responses

**Figure 2:**
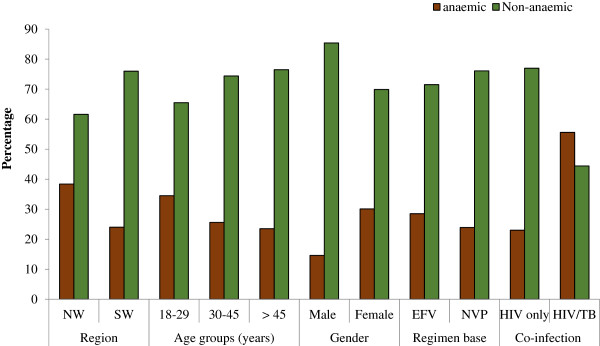
Prevalence of anaemia [Hgb < 11 g/dl] among study participants.

A significant difference in haematological parameters for participants from the two regions was observed only in red blood cell (RBC) counts (p < 0.001). Gender had a significant influence (p < 0.02) on mean haemoglobin (Hgb), RBC and platelet (PLT) counts except white blood cell (WBC) count (Table 2). HIV-mono infected patients had a significantly higher level of WBC (p = 0.004), PLT (p = 0.035), RBC counts and Hgb (p < 0.001) when compared with HIV/TB co-infected counterparts. On the other hand, there was no significant difference in most haematologic parameters between EFV-based and NVP-based regimens except for an increase in platelet count levels which was significantly higher (p = 0.024) among patients on EFV-based regimen (Table 3). In general, the prevalence of anaemia (Hgb < 11 g/dl), neutropenia (WBC < 2000 cells/mm^3^) and thrombocytopenia (PLT < 125 × 10^3^ cells/mm^3^) in the study participants was 26.2%, 1.6% and 5.9% respectively. Among anaemic patients, 62.3%, 25.7% and 12% had mild, moderate and severe anaemia respectively. Anaemia was significantly higher in patients from the NW region (p = 0.003) when compared to those from SW region. Similarly, females (p < 0.001) and HIV/TB co-infected patients (p < 0.001) were more anaemic when compared to males and HIV-mono-infected patients respectively (Figure 2). There was no significant difference (p = 0.959) in the prevalence of anaemia between patients on AZT-containing regimen (24.9%) compared with those not on AZT regimen (25.1%).

### Virologic responses

**Figure 3:**
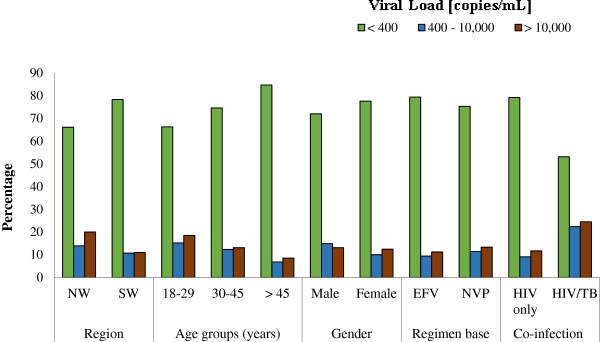
Prevalence of virologic categories among study participants.

Of the 951 participants, viral load was performed in 89.3% (849/951) and detected in 59.2%, while 40.8% of the participants had undetectable viral loads (< 40 RNA copies/mL) at median treatment duration of 24 months (Range: 1–156 months). The difference in mean viral load was insignificant (P > 0.05) between participants from the NW and SW regions (Table 2), HIV mono-infected and HIV/TB co-infected patients and in patients on EFV-based and NVP-based regimens (Table 3). However, the mean viral load was significantly different in the various age groups (p = 0.033) with the highest mean load in those 18–29 years of age (Table 2). The prevalence of virologic failure was 23.2% at median treatment duration of 16 months (Range: 6 – 132 months), while 76.8% attained adequate viral suppression at median treatment duration of 28.5 months (Range: 6 – 156 months). Definite virologic failure (> 10,000 RNA copies/mL) was 12.3% at median treatment duration of 16 months (Range: 6–132 months). Definite virologic failure was significantly prominent in the age group 18–29 years (18.5%, p = 0.013), in HIV/TB co-infected patients (24.5%, p < 0.001) and among participants from the NW region (20%, p = 0.013) (Figure 3).

Haemoglobin concentration (r = 0.208, p < 0.001), RBC (r = 0.129, p = 0.009) and WBC (r = 0.168, p = 0.001) counts correlated significantly with CD4 + T-cell counts. Both haematologic and CD4 + T-cell counts showed a negative correlation with viral load although not statistically significant (p > 0.05). Generally, mean Hgb and CD4 counts improved with ART duration, while mean viral load decreased.

### Factors associated with virologic failure

In a univariate analysis, socio-demographic (Table 4) and behavioural variables (Table 5) except age did not have any significant influence on the prevalence of virologic failure. Failure was associated with the age groups 18–28 years (OR 2.81, 95% CI: 1.55 – 5.10) and 30–45 years (OR 1.95, 95% CI: 1.21 – 3.13). Similarly, patients from the NW region had a higher odds (OR 1.79, 95% CI: 1.16 – 2.76) of experiencing virologic failure than those from SW region. Distance from treatment centres was also an important determinant of virologic failure. Patients living out of the municipality were more likely to experience virologic failure (OR 2.30, 95% CI: 1.53 – 3.46) than their counterparts residing within the municipality (Table 4).

**Table 4:** Demographic and socio-economic factors associated with virologic failure and risk estimates

**Factors Demographic**	**Virologic responses: prevalence, n (%)**				**Risk estimates (95% CI)**		
	**N**	**VF**	**AS**	**p-value**	**Crude odd ratio**	**Adjusted odd ratio**	**p-value**
**Gender:**							
Male	175	46 (26.3)	129 (73.7)	0.260	1.26 (0.84-1.87)		
Female	502	111 (22.1)	391 (77.9)		1		
**Age (years):**							
18 – 29	96	31 (32.3)	65 (67.7)	0.002	2.81 (1.55-5.10)	0.27 (0.10-0.83)	0.024
30 – 45	402	100 (24.9)	302 (75.1)		1.95 (1.21-3.13)	0.44 (0.20-1.07)	0.079
> 45	179	26 (14.5)	153 (85.5)		1	1	
**Region:**							
Northwest	120	39 (32.5)	81 (67.5)	0.008	1.79 (1.16-2.76)	0.20 (0.10-0.74)^*^	
Southwest	557	118 (21.2)	439 (78.8)		1	1	
**Distance to TC:**							
Out of municipality	281	80 (28.5)	201 (71.5)	< 0.001	2.30 (1.53-3.46)	0.42 (0.20-0.94)	0.011
Within municipality	312	46 (14.7)	266 (85.3)		1	1	
**Socio-economic**							
**Level of education**							
Low (≤ 7yrs. of school)	273	46 (16.8)	227 (83.2)	0.118	0.72 (0.47-1.09)		
High (> 7 yrs. of school)	309	68 (22.0)	241 (78.0)		1		
**Income level:**							
≤ 50,000 XAF ^ɖ^	480	94 (19.6)	386 (80.4)	0.960	0.99 (0.58-1.69)		
> 50,000 XAF	101	20 (19.8)	81 (80.2)		1		
**Marital status:**							
Never married	167	45 (25.7)	124 (74.3)		1.57 (0.92-2.69)		
Currently married	245	42 (17.1)	203 (82.9)	0.079	0.94 (0.55-1.59)		
Previously married	155	28 (18.1)	127 (81.9)		1		

^*^ Model including region of residence and excluding HIV/TB co-infection, VF: Virologic failure (viral load > 400 RNA copies/mL after 6 months of ART), AD: Adequate suppression (≤ 400 RNA copies/mL after 6 months of treatment), TC: treatment centre, CI- confidence interval, ^ɖ^ 50,000 XAF = ~ 100 US dollars.

**Table 5:** Behavioural and clinical factors associated with virologic failure and risk estimates

**Factors Behavioural**	**Virologic responses: prevalence, n (%)**				**Risk estimates (95% CI)**		
	**N**	**VF**	**AS**	**p-value**	**Crude odd ratio**	**Adjusted odd ratio**	**p-value**
**Alcohol:**							
Yes	313	65 (20.8)	248 (79.2)	0.927	0.98 (0.66-1.46)		
No	280	59 (21.1)	221 (78.9)		1		
**Smoking:**							
Yes	55	15 (27.3)	40 (72.7)	0.223	1.48 (0.79-2.77)		
No	538	109 (20.3)	429 (79.7)		1		
**Clinical history**							
**Duration of ART**							
(months): ≤ 12	108	48 (44.4)	60 (55.6)	< 0.001	4.24 (2.49-7.23)	1.11 (0.40-3.16)	0.851
12-36	361	76 (21.1)	285 (78.9)		1.41 (0.90-2.22)	1.57 (0.73-3.36)	0.251
> 36	208	33 (15.9)	175 (84.1)		1	1	
**Regimen type:**							
EFV-based	177	35 (19.8)	142 (80.2)	0.232	0.77 (0.51-1.18)		
NVP-based	492	119 (24.2)	373 (75.8)		1		
**Co-infection status:**							
HIV/TB co-infected	51	22 (45.1)	28 (54.9)	< 0.001	3.24 (1.80-5.84)	0.20 (0.04-0.70)	0.014
HIV only	589	119 (20.2)	470 (79.8)		1	1	
**CD4+T-cell (cells/μL):**							
< 200	154	46 (29.9)	108 (70.1)	0.002	2.49 (1.0-6.31)	0.68 (0.23-2.05)	0.497
200 - 499	165	24 (14.5)	141 (85.5)		1.69 (0.63-4.49)	1.22 (0.40-3.75)	0.724
≥ 500	41	06 (14.6)	35 (85.4)		1	1	
**Anaemic status (g/dL):**							
Anaemic	114	34 (29.8)	80 (70.2)	< 0.001	2.48 (1.52-4.07)	2.30 (1.10-4.83)	0.028
Non-anaemic	376	55 (14.6)	321 (85.4)		1	1	

VF: Virologic failure (viral load > 400 RNA copies/mL after ≥ 6 months of ART), AS: Adequate suppression (≥ 400 RNA copies/mL after ≥ 6 months of ART), Anaemia: Hgb < 11 g/dl, CI - confidence interval.

Similarly, duration of ART, co-infection with TB, CD4 + T-cell count and anaemic status were associated with virologic failure. HIV/TB co-infected patients had remarkably higher odds (OR 3.24, 95% CI: 1.80 – 5.84) of experiencing virologic failure than HIV mono-infected patients, while patients on ART for < 12 months had higher odds (OR 4.24, 95% CI 2.49 – 7.23) than those who have been on ART for 12–36 months and > 36 months. Similarly, anaemic patients (OR 2.48, 95% CI: 1.52 – 4.07) and patients with CD4+ T-cell count < 200 cells/μL (OR 2.49, 95% CI: 1.0 – 6.31) were also prone to failing ART. Nonetheless, there was no significant difference in virologic failure between those on NVP-based and EFV-based regimen (Table 5).

In a multivariate analysis, involving all significantly associated variables except region of residence, the age group 18–29 years (AOR 0.27, 95% CI: 0.10 – 0.83), co-infection with TB (AOR 0.20, 95% CI: 0.04– 0.70) and anaemic patients (AOR 2.30, 95% CI: 1.10 – 4.83) were again associated with virologic failure. Another model which included all significant variables except HIV/TB co-infection, revealed that the age group 18 – 29 years (p = 0.022), anaemia (p = 0.024) and distance from treatment centre (p = 0.011) with the same adjusted odd ratios as in the previous model were associated with virologic failure.

## Discussion

Diminution of CD4 + T-cells and haematological complications are hallmarks of HIV disease progression [15] associated with increase morbidity and mortality [16]. The incidence and severity of these factors generally correlate with the stage of the disease with anaemia being the most common and important haematologic predictor of HIV progression to AIDS [17]. In combination with other clinical markers, immuno-haematologic parameters are used routinely in the evaluation and monitoring of HIV-infected persons especially in resource limited settings. They are relatively reliable indicators of prognosis that complement the viral load assay and therefore guide therapeutic decisions regarding antiretroviral treatment [18]. On the other hand, peak viral levels in adults are not predictive of the rate of disease progression per se. However viral set point, which is most likely a measure of the dynamics between the virulence of the virus strain and the ability of the host immune system to contain the virus, is highly predictive of disease progression [19].

In the present study, there was generally, an overall improvement in the means of immunologic and hematologic parameters of study participants with ART duration. The means of all haematologic parameters except that for platelet counts were significantly higher (p < 0.001) in patients who at recruitment were on ART for > 36 months. This increase in immuno-hematologic parameters corresponded with a significant decrease in mean viral loads in participants as ART duration increased (Table 3). A rise in CD4 + T-cell counts significantly correlated with increase values of Hgb (p < 0.001), RBC (p < 0.009) and WBC (p = 0.001) and these constitute indicators of improved treatment outcome. These findings corroborate previous studies that demonstrated an improvement in the haematological and immunological parameters as well as reduced morbidity and mortality in HIV infected persons who had been on HAART for a long duration [20,21].

There was also a demographic disparity in immuno-haematologic parameters. Over 70% of participants in the current study were females. Previous studies have also reported a high female population as well as the fact that females are infected early in life than males [22], [23]. Females have also been observed to have a better health-seeking attitude than males [24]. Our results show that increase in age had a better prediction in terms of immuno-haematologic and virologic responses to ART. Furthermore, the mean CD4 + T-cell count of HIV/TB co-infected patients was 1.6 times lower than that of HIV mono-infected persons. Co-infected patients were also more anaemic than HIV mono-infected patients, which is in line with a previous report by Kufa et al. [25]. CD4 + T-cell-count of < 200 cells/μL was predominant among HIV/TB co-infected patients (Figure 1). In a previous study, CD4 + T-cell count < 200 cells/μL was associated with an increased risk of anaemia [26].

Mean viral load and CD4 + T-cell counts did not differ between males and females, although prior studies [27,28] suggest a significant influence of gender on viral dynamics and the immune response. In a recent report, women showed consistently better immune responses to treatment than did men in a virally suppressed population [29]. However, in our study, females were more at risk of developing anaemia compared with males (p < 0.001) which is in line with findings by Levine et al. [30] who attributed this difference to sex and race. Similar findings by Volberding et al. [31] attributed anaemia to menstrual blood loss in women and to the drain on iron stores that occur with pregnancy and delivery. Even with the use of HAART, anaemia remains strongly and consistently associated with HIV disease progression [32]. Although the prevalence of severe anaemia has decreased since the introduction of HAART, mild-to-moderate anaemia continues to be common [33]. In this study, 61.3%, 26.9% and 11.8% of anaemic patients suffered from mild, moderate and severe anaemia respectively. Our study did not show any significant difference in the occurrence of anaemia between patients on zidovudine-containing regimens and those without as also reported by Semba et al. [34].

In line with previous reports our results further indicated that there was no significant difference in mean CD4 count [35] and mean viral load [36,37] in those treated with NVP-based and EFV-based regimens, in contrast to another report [38] that showed a better outcome with Efavirenz-based ART compared with Nevirapine. The lower mean immuno-hematologic parameters seen in participants from the NW when compared with those from the SW region was probably because most of the HIV/TB co-infected patients were from the NW region. Moreover, our results showed HIV co-infection with TB was associated with poor immunologic and hematologic responses.

Attaining and maintaining viral load to an undetectable level is the key to mitigating long-term risk of AIDS-defining illnesses among HIV-positive patients with low CD4 counts. Patients who are initiated on ART and experience rapid CD4 increase, have a lower risk of AIDS illness than those with a slow response. Nonetheless, this difference diminishes after six months provided that viral load remains undetectable [39]. At a median treatment duration of 24 months, 40.8% of the study participants had undetectable viral loads with an overall 76.8% adequate viral suppression rate at a median treatment duration of 28.5 months. The prevalence of virologic failure was 23.2% of which 12.3% reflected a definite virologic failure. These rates are similar to those reported in other resource-limited settings [40] and comparable to those reported in developed countries. In spite of earlier incertitude, this shows that ART programs based on the public health approach using similar regimens in resource-limited countries, perform as effective as those seen in clinical cohorts in developed countries [41-43].

Even though socioeconomic status has been associated with HIV treatment outcomes [44] our study surprisingly did not suggest an association between socio-economic or behavioural predictors of virologic failure. However, participants from the NW region, and patients residing far from treatment centres were more likely to experience virologic failure. Previous studies and extensive reviews on barriers to accessing HIV treatment and negative treatment outcomes suggest that longer distances from treatment sites are associated with poorer outcomes [45,46].

The higher occurrence of virologic failure in the younger age group of 18–29 years corroborates a previous report by Anude et al. [47] and this can be attributed mainly to non-adherence to treatment among the youths [46]. Other studies have also demonstrated that improved ART outcomes increases with age [48]. Similarly, virologic failure was common among HIV/TB co-infected and anaemic patients as well as in patients with CD4 + T-cell counts of < 200 cell/μL. Studies conducted by Skowron et al. [49] demonstrated that CD4 + T-cell count is a better predictor of viral suppression. Likewise, Bello et al. [50] showed that TB co-morbidity had a significant influence on the occurrence of virologic failure by accelerating the course of HIV induced disease resulting from CD + T-cells decline.

## Conclusions

This study demonstrates a favourable immuno-haematologic and virologic outcome following antiretroviral therapy. Anaemia, co-infection with TB, the age group 18–29 years and distance from the treatment centre independently predicted virologic failure. Despite this favourable outcome, it is imperative for the National AIDS Control Committee (NACC) to step-up efforts to improve on ARV distribution, proper assessment and management of anaemia, early diagnosis and treatment of TB and treatment adherence counselling especially in younger patients.

## Abbreviations

ART: Antiretroviral therapy; HAART: Highly active antiretroviral therapy; ARV: Antiretroviral; AZT: Zidovudine; NVP: Nevirapine; EFV: Efavirenz; 3TC: Stavudine; SW: Southwest; NW: Northwest; NACC: National AIDS control committee.

## Competing interests

The authors declare that they have no competing interests.

## Authors’ contributions

Conceived and designed the experiments: HDM, TN-A and FC-N. Sample collection and Laboratory analysis: HDM, KAT, MHA and BAN. Analysed the data: HDM and PNA. Contributed reagents/materials: HDM, TN-A, and FC-N. Wrote the paper: HDM, PNA, FC-N and TNA. All authors read and approved the final manuscript.

## Supplementary Material

Additional file 1 Multilingual abstracts in the six official working languages of the United Nations. Click here for file
