# Supplementary material for: Immuno-haematologic and virologic responses and predictors of virologic failure in HIV-1 infected adults on first-line antiretroviral therapy in Cameroon
Source: Infect Dis Poverty. 2014 Jan 30;3:5. doi: 10.1186/2049-9957-3-5 (PMC3922096; doi:10.1186/2049-9957-3-5)
Supplement: Additional file 1 — Multilingual abstracts in the six official working languages of the United Nations. [file 2049-9957-3-5-S1.pdf]

Translation of the abstract into the six official working languages of the United Nations

التجاوبات الدموية المناعية والفيروسية والمنبئات بالفشل الفيروسي لدى البالغين المصابين بعدوى فيروس العوز المناعي البشري-1 والموضوعين على علاج بالخط الأول من مضادات الفيروسات القهقرية في الكاميرون

هنري د ميريكي، كوكوا أ توفون، مبونكا هـ أفيغينوي، برنارد أ نيندم، باسكال ن أتانغا، داميان ن أنونغ، فيديليس تشو-نغوا، تيريزا نكو-أكنجي

الملخص

الخلفية: هناك حاجة للحصول على بيانات متزامنة عن التجاوبات المناعية، والدموية والفيروسية وعن المنبئات بالفشل الفيروسي بعد بدء العلاج المجاني بمضادات الفيروسات القهقرية في الكاميرون، وذلك من أجل تقييم خوارزميات مراقبة العلاج الحالي، ولإكمال الجهود تحسين وتطوير تدبير حالات العدوى بفيروس العوز البشري.

الطرق: كانت هذه دراسة مستعرضة أجريت بين أكتوبر 2010 ويونيو 2012. بلغ العدد الإجمالي من الذين أدرجوا في هذه الدراسة 951 مشارك تراوحت أعمارهم بين 18 و 74 سنة من مراكز مختارة حاصلة الموافقة لعلاج فيروس العوز المناعي البشري من المناطق الشمالية الغربية والجنوبية الغربية من الكاميرون. هذه العينة من 247 رجل و704 امرأة. تم الحصول على البيانات الديمغرافية والسلوكيات الخطرة المبلغ عنها والبيانات الاقتصادية الاجتماعية بواسطة استبيان منظم. أجري تعداد الدم الكامل والخلايا التائية الايجابية CD4 بالوسائل التقنية القياسية الأوتوماتيكية. وجرى تقدير الحمل الفيروسي باستخدام نظام Abbott Real*Time* HIV-1 *m*2000™. تم تحليل البيانات باستخدام برنامج SPSS version 17. وبلغ مستوى الدلالة الإحصائية P □0.05.

النتائج: بلغت المدة الوسطية للعلاج المضاد للفيروسات القهقرية 24 شهرا. وبلغ وسطي عدد الخلايا التائية الايجابية CD4 لدى المجموعة المدروسة 255.3 خلية/ميكرولتر [فترة الثقة 95%، 236.8 – 273.9]. وبشكل إجمالي، كان عدد الخلايا التائية الايجابية CD4 لدى 45.9%، 43.8% و10.2% من المشاركين □ 200 خلية/ميكرولتر، 200 – 499 خلية/ميكرولتر و□ 500 خلية/ميكرولتر، على التوالي. كان فقر الدم (الأنيميا) موجودا لدى 26.2% من المشاركين، وُصِف 62.3%، 25.7% و 12% منها بأنه خفيف، متوسط أو شديد، على التوالي. حدث الفشل الفيروسي لدى 23.2% من المشاركين وكان الحمل الفيروسي لدى12.3% منهم □ 10,000 نسخة RNA/ مللتر. في نفس الوقت حصل 76.8% من المرضى على قمع فيروسي كاف ووصل 40.8% منهم إلى معدل حمل فيروسي لا يمكن كشفه. أما الفئة العمرية 18-29 عاما (p = 0.024)، ووجود إصابة مشاركة بالسل (p = 0.014)، وفقر الدم (p = 0.028) والبعد عن مركز العلاج (p = 0.011) فقد تم توقع حدوث الفشل الفيروسي فيها بشكل مستقل.

الاستنتاج: حققت الغالبية العظمى من المشاركين قمعا فيروسيا كافيا بعد $\leq$ ستة أشهر من العلاج بمضادات الفيروسات القهقرية. وعلى الرغم من هذه النتائج الدموية المناعية والفيروسية المناسبة، يجب أن يكثف البرنامج

الوطني لمكافحة الإيدز جهوده الرامية إلى تحسين توزيع الأدوية المضادة للفيروسات القهقرية، فضلا عن إجراء التقييم السليم لفقر الدم وعلاجه، ودعم التشخيص المبكر لمرض السل وعلاجه وتعزيز استشارات الالتزام بالعلاج، وخاصة بين المرضى الأصغر سنا.

Translated from English version into Arabic by Lina SM, through

TRANSLATORS
WITHOUT BORDERS

喀麦隆艾滋病感染者对一线抗逆转录病毒治疗的免疫血液学与病毒学反应和病毒学治疗失败的预测因子

Henry D Meriki, Kukwah A Tufon, Mbunkah H Afegenwi, Bernard A Nyindem, Pascal N Atanga, Damian N Anong, Fidelis Cho-Ngwa, Theresa Nkuo-Akenji

摘要

引言：在喀麦隆，艾滋病感染者在接受最初的免费抗病毒治疗后，常常会发生病毒学治疗失败。收集艾滋病感染者病毒学治疗失败后相关的免疫学、血液学和病毒学证据对评估和监测抗病毒治疗效果、促进对艾滋病感染者的管理具有重要意义。

方法：2010年10月到2012年6月，我们在喀麦隆西北和西南区域的艾滋病治疗中心招募了951名正在接受抗病毒治疗的艾滋病感染，其中男性247名，女性704名，年龄在18至74岁之间。通过问卷调查收集社会人口学，社会经济学和生活行为习惯方面的资料。全血和CD4+T淋巴细胞计数按标准的自动化技术检测。病毒载量（VL）检测方法采用Abbott Real*Time* HIV-1 *m*2000™ 检测系统。数据分析采用SPSS 17.0软件，检验水准为 $\alpha = 0.05$。

结果：所有参与者接受抗病毒治疗的平均时间（中位数）是24个月，CD4+T淋巴细胞计数平均为255.3 个/μL [95% *CI*, 236.8–273.9]. 其中45.9%的艾滋病感染者 $CD4^+$ T淋巴细胞计数小于200个/μL , 43.8%的艾滋病感染者CD4+T淋巴细胞计数在200-499个/μL之间，10.2%的艾滋病感染者CD4+T淋巴细胞计数大于500 个/μL。贫血患者占26.2%，其中轻度、中度和重度贫血的比例分别为62.3 %、25.7% 和12%。病毒学治疗失败发生率为23.2% ，其中12.3%的艾滋病感染者病毒载量大于10000拷贝/mL。有76.8% 艾滋病感染者达到病毒抑制，其中40.8%的艾滋病感染者检测不到病毒载量。18-29岁年龄组($p = 0.024$)、合并肺结核感染 ($p = 0.014$)、贫血($p = 0.028$) 和居住地距离抗病毒治疗中心较远(p = 0.011)是抗病毒治疗病毒学治疗失败的危险因素。

结论： 多数艾滋病感染者在接受六个月以上的抗病毒治疗后，艾滋病病毒都得到有效抑制。除了要监测免疫血液学和病毒学指标外，国家艾滋病控制项目应当努力增加抗病毒治疗药物的合理分配和评估，并加强对贫血和结核病的早期诊断和治疗，加强对艾滋病感染者，尤其是年轻感染者的抗病毒治疗的咨询服务。

Translated from English version into Chinese by TIAN Li-guang, edited by YU Sen-hai, through

CDC
CHINESE CENTER FOR DISEASE CONTROL AND PREVENTION
NATIONAL INSTITUTE FOR PARASITIC DISEASES

Réponses immunohématologiques et virologiques et éléments prédictifs de l'échec virologique chez les adultes infectés par le VIH-1 sous traitement antirétroviral de première ligne au Cameroun

Henry D. Meriki, Kukwah A. Tufon, Mbunkah H. Afegenwi, Bernard A. Nyindem, Pascal N. Atanga, Damian N. Anong, Fidelis Cho-Ngwa, Theresa Nkuo-Akenji

Résumé

Contexte : Des données contemporaines sur les réponses immunologiques, hématologiques et virologiques et sur les éléments prédictifs de l'échec virologique suite à la mise en place d'un traitement antirétroviral gratuit sont nécessaires pour évaluer l'algorithme actuel de suivi du traitement et compléter les efforts d'amplification et d'amélioration de la lutte contre les infections par le VIH au Cameroun.

Méthodologie : Cette étude transversale a été menée entre octobre 2010 et juin 2012. Au total, 951 participants âgés de 18 à 74 ans (247 hommes et 704 femmes) ont été recrutés dans des centres de traitement du VIH agréés dans les région Nord-ouest et Sud-ouest du Cameroun. Les données démographiques et socioéconomiques et les comportements à risque rapportés par les sujets ont été relevés à l'aide d'un questionnaire structuré. La formule sanguine et la numération des lymphocytes T CD4+ ont été obtenues par des méthodes automatisées standard. La charge virale (VL) a été déterminée à l'aide d'un système Abbott Real*Time* HIV-1 *m*2000™. Les données ont été analysées avec SPSS version 17. Le niveau de signification statistique était de $P < 0{,}05$.

Résultats : La durée médiane du traitement antirétroviral (TAR) était de 24 mois. La numération moyenne de CD4+ dans la population était de 255,3 cellules/µl (IC à 95 % 236,8–273,9). Dans l'ensemble, la numération de CD4+ était de < 200 cellules/µl chez 45,9 % des participants, 200 à 499 cellules/µl chez 43,8 % et > 500 cellules/µl chez 10,2 %. Une anémie a été observée chez 26,2 % des participants ; elle était décrite comme légère chez 62,3 % d'entre eux, modérée chez 25,7 % et sévère chez 12 %. L'échec virologique a été constaté chez 23,2 % des participants, dont 12,3 % avaient une charge virale supérieure à 10 000 copies d'ARN/ml. En revanche, 76,8 % des participants ont atteint une suppression virale adéquate, avec une charge virale indétectable chez 40,8 %. L'appartenance au groupe d'âge de 18 à 29 ans ($p = 0{,}024$), la co-infection par la tuberculose ($p = 0{,}014$), l'anémie ($p = 0{,}028$) et l'éloignement du

centre de traitement (p = 0,011) étaient prédictifs de l'échec virologique, indépendamment les uns des autres.

Conclusion : La majorité des participants a atteint une suppression virale adéquate après au moins six mois de TAR. Malgré les résultats immunohématologiques et virologiques favorables, le Programme national de lutte contre le SIDA aurait intérêt à intensifier ses efforts d'amélioration de la distribution d'antirétroviraux et à mettre en place une évaluation et une prise en charge adéquates de l'anémie, à encourager le diagnostic et le traitement précoces de la tuberculose et à intensifier le conseil sur l'observance, en particulier auprès des patients jeunes.

Translated from English version into French by Suzanne Assenat, through

TRANSLATORS
WITHOUT BORDERS

Иммуногематологический и вирусологический ответы и прогностические факторы вирусологической неэффективности лечения среди взрослых, инфицированных ВИЧ-1, проходящих антиретровирусную терапию первой линии, в Камеруне.

Генри Д. Мерики, Куква А. Туфон, Мбунка Х. Афегенви, Бернард А.Нйиндем, Паскаль Н. Атанга, Дамиан Н. Анонг, Фиделис Чо-Нгва, Тереза Нкуо-Акенджи

Резюме

Предпосылки исследования: Современные данные относительно иммунологических, гематологических и вирусологических ответов и прогностических факторов вирусологической неэффективности после начала бесплатной антиретровирусной терапии в Камеруне необходимы для оценки существующего алгоритма мониторинга терапии, а также в качестве дополнения к действиям, направленным на увеличение масштаба и улучшение терапии ВИЧ-инфекций.

Методы исследования: Данное исследование охватывало различные группы населения и проводилось с октября 2010 года по июнь 2012 года. Общее число участников составляло 951 человек в возрасте от 18 до 74 лет, отобранных в определённых проверенных центрах ВИЧ-терапии в северо-западных и юго-западных регионах Камеруна. В исследовании принимали участие 247 мужчин и 704 женщины. С помощью структурированной анкеты были получены демографические и социально-экономические данные, а также те данные, касающиеся форм поведения, повышающих риск заражения ВИЧ, которые сообщили сами участники исследования. Полный анализ крови и определение количества Т-лимфоцитов CD4+ проводились с использованием стандартных автоматизированных методов. Определение вирусной нагрузки (VL) проводилось с использованием системы Abbott Real*Time* HIV-1 *m*2000™. Данные были проанализированы с помощью SPSS version 17. Статистический уровень значимости составлял менее 0,05.

Результаты: Средняя продолжительность антиретровирусной терапии (ART) составляла 24 месяца. Среднее по совокупности количество Т-лимфоцитов CD4+ составляло 255,3 клеток/мкл [доверительный интервал 95%, 236.8–273.9]. Количество Т-лимфоцитов CD4+ составляло менее 200 клеток/мкл, от 200 до 499 клеток/мкл и более 500 клеток/мкл у 45,9%, 43,8% и 10,2% участников

соответственно. Анемия была зафиксирована у 26,2% участников: лёгкая, средняя и тяжёлая у 62,3%, 25,7% и у 12% соответственно. Вирусологическая неэффективность была зафиксирована у 23,2% участников, при этом у 12,3% участников уровень вирусной нагрузки составлял более 10 000 копий РНК/мл. В то же время 76,8% пациентов достигли адекватной вирусной супрессии, при этом у 40,8% определить вирусную нагрузку было невозможно. Возрастная группа 18 – 29 лет ($p = 0.024$), коинфекция тубекулёза ($p = 0.014$), анемия ($p = 0.028$) и удалённость от лечебного центра ($p = 0.011$) независимо предсказали вирусологическую неэффективность.

Заключение: Большинство участников достигли достаточной вирусной супрессии после шести или более месяцев ART. Несмотря на эти положительные иммуногематологические и вирусологические результаты, Национальная программа по контролю за распространением СПИДа должна увеличить усилия, направленные на улучшение распространения антиретровирусных препаратов, а также усилить меры по правильной оценке масштабов распространения анемии и борьбы с ней, по способствованию ранней диагностике и лечению тубекулёза и повышению эффективности консультирования с целью объяснения важности точного соблюдения режима лечения, особенно среди более молодых пациентов.

Translated from English version into Russian by MariyaN, through

TRANSLATORS
WITHOUT BORDERS

Respuestas inmunohematológicas y virales y predictores de falla viral en adultos infectados con VIH-1 en el tratamiento antirretrovírico de primera línea en Camerún

Henry D Meriki, Kukwah A Tufon, Mbunkah H Afegenwi, Bernard A Nyindem, Pascal N Atanga, Damian N Anong, Fidelis Cho-Ngwa, Theresa Nkuo-Akenji

Resumen

Antecedentes: Se necesitan datos contemporáneos sobre las respuestas inmunológicas, hematológicas y virológicas y los predictores de falla viral luego del inicio del tratamiento antirretrovírico en Camerún para evaluar el algoritmo de monitoreo del tratamiento utilizado actualmente, así como para complementar los esfuerzos por ampliar y mejorar el control de las infecciones por VIH.

Métodos: Este fue un estudio de corte transversal llevado a cabo entre octubre de 2010 y junio de 2012. Se tomó un total de 951 participantes entre los 18 y 74 años de centros aprobados de tratamiento de VIH en las regiones noroeste y suroeste de Camerún, compuesto de 247 pacientes masculinos y 704 femeninos. Los datos demográficos y socioeconómicos y los comportamientos de riesgo auto-reportados se obtuvieron a través de un cuestionario estructurado. Los recuentos de glóbulos y de células T CD4+ se realizaron con técnicas automatizadas estándar. La determinación de la carga viral (VL) se realizó utiliando el sistema Abbott Real*Time* HIV-1 *m*2000™. Los datos se analizaron utilizando SPSS versión 17. El nivel de significación estadística fue de $P < 0,05$.

Resultados: La duración mediana del tratamiento antirretrovírico (ART) fue de 24 meses. El recuento de células T CD4+ promedio para la población fue de 255,3 célulass/µL [95% CI, 236,8–273,9]. En general, 45,9%, 43,8% and 10,2% de los participantes tenían recuentos de células T CD4+ de < 200 células/µL, 200–499 célulass/µL y > 500 células/µL, respectivamente. Se presentó anemia en 26,2% de los participantes con 62,3 %, 25,7% y 12% descritas como leve, moderada y grave, respectivamente. Hubo falla viral en 23,2% de los participantes con 12,3% con VL > 10,000 copias de RNA/mL. El restante 76,8% de los pacientes llegó a una supresión viral adecuada, con 40,8% con una carga viral indetectable. El grupo etario de 18 a 29 años ($p = 0.024$), co-infectado por tuberculosis ($p = 0.014$), con anemia ($p = 0.028$) y distancia del centro de tratamiento ($p = 0.011$) predijo independientemente la falla viral.

Conclusión: La mayoría de los participantes logró una supresión viral adecuada luego de ≥ seis meses de ART. A pesar de estos resultados inmunohematológicos y virológicos favorables, el National AIDS Control Programme debería renovar sus esfuerzos en la mejora de la distribución de medicamentos antirretrovíricos, además de llevar a cabo evaluaciones y tratamientos adecuados para la anemia, propiciar el diagnóstico temprano y el tratamiento de la tuberculosis y mejorar el servicio de asesoramiento para la adherencia al tratamiento, especialmente en pacientes jóvenes.

Translated from English version into Spanish by Aldana Gómez Ríos, through

TRANSLATORS
WITHOUT BORDERS
